# Supplementary material for: Three-body correlations in nonlinear response of correlated quantum liquid
Source: Nat Commun. 2021 May 28;12:3233. doi: 10.1038/s41467-021-23467-4 (PMC8163827; doi:10.1038/s41467-021-23467-4)
Supplement: Supplementary file 1 — Supplementary Information [file 41467_2021_23467_MOESM1_ESM.pdf]

Supplementary Information for
Three-body correlations in nonlinear response of correlated quantum liquid

Tokuro Hata*,[1] Yoshimichi Teratani,[2] Tomonori Arakawa,[1,3] Sanghyun Lee,[1] Meydi Ferrier,[1,4] Richard Deblock,[4] Rui Sakano,[5] Akira Oguri,[2,6] and Kensuke Kobayashi†[1,7,8]

[1] *Graduate School of Science, Osaka University, Toyonaka, Osaka 560-0043, Japan.*
[2] *Department of Physics, Osaka City University, Osaka 558-8585, Japan.*
[3] *Center for Spintronics Research Network, Osaka University, Toyonaka, Osaka 560-8531, Japan.*
[4] *Université Paris-Saclay, CNRS, Laboratoire de Physique des Solides, 91405, Orsay, France.*
[5] *The Institute for Solid State Physics, The University of Tokyo, Chiba 277-8581, Japan.*
[6] *Nambu Yoichiro Institute of Theoretical and Experimental Physics, Osaka City University, Osaka 558-8585, Japan.*
[7] *Institute for Physics of Intelligence and Department of Physics, The University of Tokyo, Bunkyo-ku, Tokyo 113-0033, Japan.*
[8] *Trans-scale Quantum Science Institute, The University of Tokyo, Bunkyo-ku, Tokyo 113-0033, Japan.*

SUPPLEMENTARY NOTE 1

Two- and three body correlations in correlated quantum liquid [1]

Let us consider a quantum dot (QD) with a single electron energy $\varepsilon_\sigma = \varepsilon - \sigma\mu_{\rm B}B$, where σ is a spin σ ($=\uparrow,\downarrow$ or $+,-$). $\mu_{\rm B}$ is the Bohr magneton. The g-factor of electrons is taken as 2. $\varepsilon = -U/2$ and $B = 0$ when the QD holds time-reversal symmetry (TRS) and particle-hole symmetry (PHS) [see the center of Fig. 1c in the main text]. For the Hamiltonian ($\mathcal{H}$) of this system, the occupation number of the impurity state $\langle n_\sigma\rangle$ at temperature T is given using the free energy $\Omega \equiv -k_{\rm B}T\ln[{\rm Tr}e^{-\mathcal{H}/k_{\rm B}T}]$ such that $\langle n_\sigma\rangle = \partial\Omega/\partial\varepsilon_\sigma$ ($k_{\rm B}$ is the Boltzmann constant). The Friedel sum rule relates the phase shift δ_σ to the occupation number; $\langle n_\sigma\rangle \to \delta_\sigma/\pi$ at $T \to 0$.

Now, following Ref. [1], we define the susceptibility as:

$$\chi_{\sigma_1\sigma_2} \equiv -\frac{\partial^2\Omega}{\partial\varepsilon_{\sigma_1}\partial\varepsilon_{\sigma_2}} = -\frac{\partial\langle n_{\sigma_1}\rangle}{\partial\varepsilon_{\sigma_2}}. \tag{1}$$

The Kondo temperature and the Wilson ratio are described by the susceptibility: $T_{\rm K}^* \equiv 1/(4\sqrt{\chi_{\uparrow\uparrow}\chi_{\downarrow\downarrow}})$ and $R \equiv 1 - \chi_{\uparrow\downarrow}/\sqrt{\chi_{\uparrow\uparrow}\chi_{\downarrow\downarrow}}$ [2, 3]. Note that $k_{\rm B}T_{\rm K}^* = 1/(4\chi_{\uparrow\uparrow})$ and $R = 1 - \chi_{\uparrow\downarrow}/\chi_{\uparrow\uparrow}$ when either $\epsilon = -U/2$ or $B = 0$ because of $\chi_{\uparrow\uparrow} = \chi_{\downarrow\downarrow}$. The conventional charge and spin susceptibilities are given by the linear combinations of $\chi_{\sigma_1\sigma_2}$; $\chi_C = \chi_{\uparrow\uparrow}+\chi_{\downarrow\downarrow}+\chi_{\uparrow\downarrow}+\chi_{\downarrow\uparrow}$ and $\chi_s = \frac{1}{4}(\chi_{\uparrow\uparrow}+\chi_{\downarrow\downarrow}-\chi_{\uparrow\downarrow}-\chi_{\downarrow\uparrow})$, respectively. Thus, $\chi_{\sigma_1\sigma_2}$ contains sufficient information to describe the system in the linear response regime. The susceptibility can also be expressed as:

$$\chi_{\sigma_1\sigma_2} = \int_0^{1/T} d\tau\, \langle\delta n_{\sigma_1}(\tau)\delta n_{\sigma_2}\rangle\,, \tag{2}$$

where $\delta n_\sigma \equiv n_\sigma - \langle n_\sigma\rangle$.

The nonlinear susceptibility, which is the third derivative of the free energy, contributes to the next leading Fermi-liquid corrections when the energy level is away from half filling (see the right and left of Fig. 1c):

$$\chi_{\sigma_1\sigma_2\sigma_3} \equiv -\frac{\partial^3\Omega}{\partial\varepsilon_{\sigma_1}\partial\varepsilon_{\sigma_2}\partial\varepsilon_{\sigma_3}} = \frac{\partial\chi_{\sigma_2\sigma_3}}{\partial\varepsilon_{\sigma_1}}. \tag{3}$$

It can also be expressed as:

$$\chi_{\sigma_1\sigma_2\sigma_3} = -\int_0^{1/T} d\tau_3 \int_0^{1/T} d\tau_2\, \langle T_\tau\delta n_{\sigma_3}(\tau_3)\delta n_{\sigma_2}(\tau_2)\delta n_{\sigma_1}\rangle\,, \tag{4}$$

where T_τ denotes the time-ordering product.

---

* email: hata@phys.titech.ac.jp
† email: kensuke@phys.s.u-tokyo.ac.jp

Now, we consider the Kondo-correlated QD when either TRS or PHS is broken by using the following expression of differential conductance:

$$\frac{dI}{dV} = \frac{e^2}{h}\left(\sum_\sigma \sin^2\delta_\sigma\right) - \alpha_{\rm V}\left(\frac{eV}{k_{\rm B}T_{\rm K}}\right)^2 - \alpha_{\rm T}\left(\frac{\pi T}{T_{\rm K}}\right)^2 + \cdots . \tag{5}$$

Here, $T_{\rm K}$ is the Kondo temperature at the TRS and the PHS point: $T^*_{\rm K}(\varepsilon/U = -0.5, B = 0\ {\rm T})$. The phase shift is defined as:

$$\cot\delta_\sigma \equiv \frac{\varepsilon_\sigma + \Sigma_\sigma}{\gamma}, \tag{6}$$

where Σ_σ is the self-energy at $eV = 0$ and zero-frequency, and γ is the half width of energy levels, $\gamma = \Gamma/2$, in our definition.

$\alpha_{\rm V}$ consists of $\chi_{\sigma_1\sigma_2}$ and $\chi_{\sigma_1\sigma_2\sigma_3}$ as follows:

$$\begin{aligned}\alpha_{\rm V} &= \frac{2e^2}{h}\frac{(k_{\rm B}T_{\rm K})^2}{2}\sum_\sigma \frac{\pi^2}{4}\left[-\cos 2\delta_\sigma\left(\chi^2_{\sigma\sigma} + 5\chi^2_{\sigma-\sigma}\right) + \frac{\sin 2\delta_\sigma}{2\pi}\left(\chi_{\sigma\sigma\sigma} + 3\chi_{\sigma-\sigma-\sigma}\right)\right]\\ &= \frac{2e^2}{h}\frac{\pi^2}{64}\left(\frac{T_{\rm K}}{T^*_{\rm K}}\right)^2 \frac{1}{2\chi^2_{\uparrow\uparrow}}\sum_\sigma\left[-\cos 2\delta_\sigma \times \left(\chi^2_{\sigma\sigma} + 5\chi^2_{\sigma-\sigma}\right) + \frac{\sin 2\delta_\sigma}{2\pi}\left(\chi_{\sigma\sigma\sigma} + 3\chi_{\sigma-\sigma-\sigma}\right)\right]\\ &\equiv \frac{2e^2}{h}\frac{\pi^2}{64}\left(W_2 + W_3\right) \times \left(\frac{T_{\rm K}}{T^*_{\rm K}}\right)^2,\end{aligned} \tag{7}$$

where we use $k_{\rm B}T^*_{\rm K} = 1/4\chi_{\uparrow\uparrow}$. We define W_2 and W_3 as two- and three-body correlations, respectively, which we evaluate in the main text.

While it is not treated in this work, it is noted that the coefficient of $(\pi T/T_{\rm K})^2$, $\alpha_{\rm T}$, is:

$$\alpha_{\rm T} = \frac{2e^2}{h}\frac{\pi^2}{48}\left(\frac{T_{\rm K}}{T^*_{\rm K}}\right)^2 \frac{1}{2\chi^2_{\uparrow\uparrow}}\sum_\sigma\left[-\cos 2\delta_\sigma \times \left(\chi^2_{\sigma\sigma} + 2\chi^2_{\sigma-\sigma}\right) + \frac{\sin 2\delta_\sigma}{2\pi}\left(\chi_{\sigma\sigma\sigma} + \chi_{\sigma-\sigma-\sigma}\right)\right]. \tag{8}$$

The free particle (FP) model

General formulas

In the main text, we compare the experimental result with the $U = 0$ case, which we refer as the free particle (FP) model. We explain the detail of the FP model here.

The current for non-interacting electrons ($U = 0$) at zero temperature is:

$$I = \frac{e}{h}\int_{-eV/2}^{eV/2} d\omega \sum_\sigma \mathcal{T}_\sigma(\omega), \tag{9}$$

where $\mathcal{T}_\sigma(\omega)$ is transmission at frequency, ω:

$$\mathcal{T}_\sigma(\omega) = \frac{\gamma_0^2}{(\omega - \varepsilon_\sigma)^2 + \gamma_0^2}. \tag{10}$$

$2\gamma_0$ corresponds to the half width of a resonance peak. ε_σ is a single electron energy, where $\varepsilon_\sigma = \varepsilon - \sigma\mu_{\rm B}B$ with spin, σ. The g-factor of electrons is taken as 2. The differential conductance (dI/dV) is expressed as:

$$\frac{dI}{dV} = \frac{e^2}{h}\sum_\sigma \frac{1}{2}\left[\mathcal{T}_\sigma(eV/2) + \mathcal{T}_\sigma(-eV/2)\right] \tag{11}$$

$$= \frac{e^2}{h}\sum_\sigma \frac{1}{2}\left[\frac{\gamma_0^2}{(eV/2 - \varepsilon_\sigma)^2 + \gamma_0^2} + \frac{\gamma_0^2}{(eV/2 + \varepsilon_\sigma)^2 + \gamma_0^2}\right] \tag{12}$$

$$= \frac{e^2}{h}\left[\sum_\sigma \frac{1}{1 + (\varepsilon_\sigma/\gamma_0)^2}\right] - \alpha_{\rm V}^{(0)}\left(\frac{eV}{2\gamma_0}\right)^2 + {\rm O}(V^4), \tag{13}$$

where $\alpha_{\rm V}^{(0)}$ in the last line of the above equation is:

$$\alpha_{\rm V}^{(0)} \equiv \frac{e^2}{h}\sum_{\sigma}\frac{1-3(\varepsilon_\sigma/\gamma_0)^2}{\{1+(\varepsilon_\sigma/\gamma_0)^2\}^3}. \tag{14}$$

Because phase shift $\delta_\sigma^{(0)}$ at $U=0$ is given by $\cot\delta_\sigma^{(0)} = \varepsilon_\sigma/\gamma_0$ [see also Supplementary Equation (6)], the first term of Supplementary Equation (13) can be replaced by $\sin^2\delta_\sigma^{(0)}$:

$$\sin^2\delta_\sigma^{(0)} = \frac{1}{1+(\varepsilon_\sigma/\gamma_0)^2}. \tag{15}$$

When either the TRS or PHS is preserved, $\sin^2\delta_\uparrow^{(0)} = \sin^2\delta_\downarrow^{(0)}$, $\varepsilon_\uparrow^2 = \varepsilon_\downarrow^2$, and $\chi_{\uparrow\uparrow}^{(0)} = \chi_{\downarrow\downarrow}^{(0)}$. In terms of susceptibility, we get $\alpha_{\rm V}^{(0)}$ by replacing $k_{\rm B}T_{\rm K}$ with $2\gamma_0$ in Supplementary Equation (7):

$$\begin{aligned}\alpha_{\rm V}^{(0)} &= \frac{e^2}{h}\frac{\pi^2}{2}\left(W_2^{(0)}+W_3^{(0)}\right)\times(2\gamma_0)^2\times\chi_{\uparrow\uparrow}^{(0)2}\\ &= \frac{2e^2}{h}\sin^4\delta_\uparrow\left(W_2^{(0)}+W_3^{(0)}\right).\end{aligned} \tag{16}$$

$W_2^{(0)}$ and $W_3^{(0)}$ are defined as follows:

$$\begin{aligned}W_2 &= -\frac{1}{2\chi_{\uparrow\uparrow}^2}\sum_\sigma\cos 2\delta_\sigma\times\left(\chi_{\sigma\sigma}^2+5\chi_{\sigma-\sigma}^2\right)\\ &\xrightarrow[U=0]{} -\frac{1}{2}\sum_\sigma\cos 2\delta_\sigma^{(0)}\ \ (\because \chi_{\sigma-\sigma}^{(0)}=0)\\ &= \frac{1}{2}\sum_\sigma\frac{\gamma_0^2-\varepsilon_\sigma^2}{\varepsilon_\sigma^2+\gamma_0^2}\equiv W_2^{(0)}.\end{aligned} \tag{17}$$

$$\begin{aligned}W_3 &= \frac{1}{2\chi_{\uparrow\uparrow}^2}\sum_\sigma\frac{\sin 2\delta_\sigma}{2\pi}\left(\chi_{\sigma\sigma\sigma}+3\chi_{\sigma-\sigma-\sigma}\right)\\ &\xrightarrow[U=0]{} \frac{1}{2\chi_{\uparrow\uparrow}^{(0)2}}\sum_\sigma\frac{\sin 2\delta_\sigma^{(0)}}{2\pi}\chi_{\sigma\sigma\sigma}^{(0)}\ \ (\because \chi_{\sigma-\sigma-\sigma}^{(0)}=0)\\ &= -\sum_\sigma\frac{\varepsilon_\sigma^2}{\varepsilon_\sigma^2+\gamma_0^2}\equiv W_3^{(0)}.\end{aligned} \tag{18}$$

We use the following relations for the calculations:

$$\chi_{\sigma\sigma}^{(0)} = \frac{1}{\pi}\frac{\gamma_0}{\varepsilon_\sigma^2+\gamma_0^2} = \frac{\sin^2\delta_\sigma^{(0)}}{\pi\gamma_0}, \tag{19}$$

$$\begin{aligned}\chi_{\sigma\sigma\sigma}^{(0)} &\equiv \frac{\partial\chi_{\sigma\sigma}^{(0)}}{\partial\varepsilon_\sigma} = \frac{1}{\pi}\frac{-2\gamma_0\varepsilon_\sigma}{(\varepsilon_\sigma^2+\gamma_0^2)^2} = -\frac{2}{\pi\gamma_0^2}\cot\delta_\sigma^{(0)}\sin^2\delta_\sigma^{(0)}\\ &= -2\pi\cot\delta_{\sigma^{(0)}}(\chi_{\sigma\sigma}^{(0)})^2\end{aligned} \tag{20}$$

Evaluation of $\alpha_{\rm V}$ under magnetic field

Supplementary Equation (14) is written as follows in the TRS breaking regime ($B\neq 0$):

$$\alpha_{\rm V}^{(0)}(B) = \frac{2e^2}{h}\frac{1-3(\mu_{\rm B}B/\gamma_0)^2}{\{1+(\mu_{\rm B}B/\gamma_0)^2\}^3}, \tag{21}$$

showing that the magnetic field dependence of $\alpha_{\rm V}^{(0)}$ is determined by the value of γ_0 at zero field.

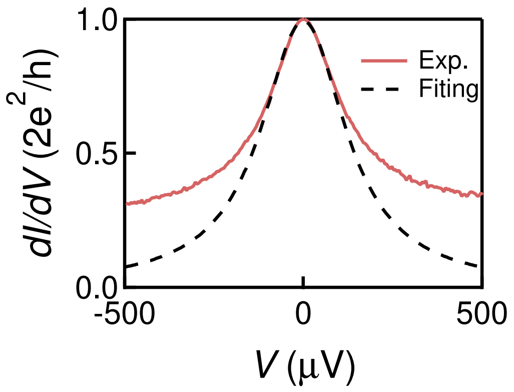

Supplementary Figure 1: The differential conductance (dI/dV) as a function of source voltage (V) at zero field.

We regard the experimental Kondo peak at zero field as if it were a resonance peak with $U = 0$ and obtain γ_0 by fitting the experimental result with the following formula:

$$\frac{dI}{dV} = \frac{2e^2}{h}\frac{\gamma_0^2}{(eV/2)^2 + \gamma_0^2}. \tag{22}$$

Supplementary Figure 1 shows the fitting result for the source-drain voltage dependence (V) of differential conductance (dI/dV) at zero field (see the detail of the experimental setup and definition of zero field in the main text). The evaluated $2\gamma_0$ is 130 μeV(= 1.5 K), which is comparable with the Kondo temperature given by the temperature dependence of the zero bias conductance (see the main text and also the next paragraph). We show the magnetic field dependence of $\alpha_{\rm V}^{(0)}$ calculated with Supplementary Equation (21) and $\gamma_0 = 65$ μeV in Fig. 3b in the main text. Similarly, we obtain the magnetic field dependence of $W_2^{(0)}$ and $W_3^{(0)}$ with:

$$W_2 = \frac{\gamma_0^2 - (\mu_{\rm B}B)^2}{(\mu_{\rm B}B)^2 + \gamma_0^2} \tag{23}$$

$$W_3 = \frac{-2(\mu_{\rm B}B)^2}{(\mu_{\rm B}B)^2 + \gamma_0^2}, \tag{24}$$

which are shown in Figs. 3a and 3c in the main text.

In our experiment, as $k_{\rm B}T_{\rm K} = 138$ μeV and $\gamma_0 = 65$ μeV, $k_{\rm B}T_{\rm K}/\gamma_0 \sim 2$ is the case. This is expected for $R \to 2$, which can be explained as follows. In the TRS and PHS case, the following is established [4],

$$\frac{dI}{dV} = \frac{2e^2}{h}\left[1 - \frac{1 + 5(R-1)^2}{4}\left(\frac{eV}{\tilde{\Gamma}}\right)^2\right]. \tag{25}$$

Here, $\tilde{\Gamma}$ is the renormalized width of the Kondo resonance, which depends on U and Γ. For $U/\Gamma \to \infty$ ($R \to 2$), $\tilde{\Gamma} = 4k_{\rm B}T_{\rm K}/\pi$ holds, and thus,

$$\frac{dI}{dV} = \frac{2e^2}{h}\left[1 - \frac{3}{2}\left(\frac{\pi}{4}\right)^2\left(\frac{eV}{k_{\rm B}T_{\rm K}}\right)^2\right]. \tag{26}$$

On the other hand, for $|eV/2\gamma_0| \ll 1$, Equation (22) yields

$$\frac{dI}{dV} = \frac{2e^2}{h}\frac{\gamma_0^2}{(eV/2)^2 + \gamma_0^2} \to \frac{2e^2}{h}\left[1 - \left(\frac{eV}{2\gamma_0}\right)^2\right]. \tag{27}$$

Comparing these two, the following is obtained.

$$\frac{k_{\rm B}T_{\rm K}}{\gamma_0} = \sqrt{\frac{3}{8}}\pi = 1.923\cdots. \tag{28}$$

It is interesting to note that, when $T_{\rm K}$ is given, the differential conductance tells us information on R in this way.

SUPPLEMENTARY NOTE 2

The analysis in the Kondo regime

Magnetic field dependence of Kondo temperature

The solid curves in Supplementary Figure 2(a) are experimental conductance at different magnetic fields. We obtain $\gamma_0(B)$ and $G_0(B)$ for each field by fitting the experimental results with Supplementary Equation (12). Supplementary Figure 2(b) shows $\mu_B B/k_B T_K$ or B dependence of $\gamma_0(0)/\gamma_0(B)$ and $G_0(B)$. T_K is the Kondo temperature at zero field, which we evaluated from the temperature dependence (see the main text).

In the $U = 0$ case, $T_K^*(B)$, $\gamma_0(B)$, and phase shift δ_σ are related as:

$$4k_B T_K^* = \frac{\pi\gamma_0(B)}{\sin^2\delta_\sigma}. \tag{29}$$

Here, T_K^* is a characteristic temperature nominally obtained according to the definition of $T_K^* \equiv 1/(4\sqrt{\chi_{\uparrow\uparrow}\chi_{\downarrow\downarrow}})$.

Thus, we obtain the following relation between T_K^* and $\gamma_0(B)$:

$$\frac{T_K^*(0)}{T_K^*(B)} = \frac{\gamma_0(0)}{\gamma_0(B)}\frac{G(B)}{G(0)}, \tag{30}$$

Thus, we get $T_K^*(0)/T_K^*(B)$ by multiplying $\gamma_0(0)/\gamma_0(B)$ and $G_0(B)$ [the red circles in Supplementary Figure 2(c). The evaluation agrees well with the theoretical curve given by NRG calculations with $U/\Gamma = 3.5$ [5].

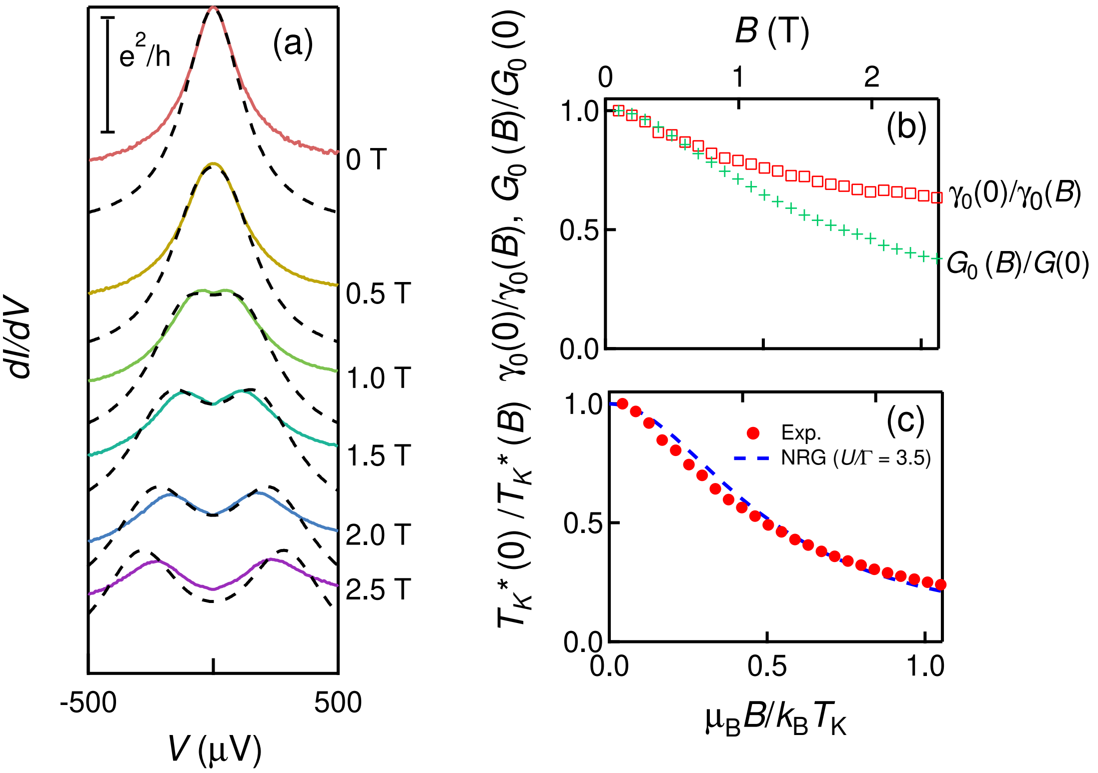

Supplementary Figure 2: (a) dI/dV as a function of V for different magnetic fields. The dashed lines are fitting curves with Supplementary Equation (12). (b) The red squares are $\gamma_0(0)/\gamma_0(B)$ obtained from the fitting. The green cross marks are the zero bias conductance. (c) The red circles are $T_K^*(0)/T_K^*(B)$, which we obtained by multiplying $\gamma_0(0)/\gamma_0(B)$ with $G(B)/G(0)$. The dashed line is given by the NRG calculations with $U/\Gamma = 3.5$.

Small dip in conductance at finite magnetic field.

Supplementary Figure 3(a) is dI/dV as a function of $eV/k_{\rm B}T_{\rm K}$ at 2 T ($\mu_{\rm B}B/k_{\rm B}T_{\rm K} = 0.84$), where Zeeman splitting (ZS) is clearly seen. There is a small dip or cusp-like structure just around the zero bias, whose shape is not parabolic to cause a sharp change in dI/dV as a function of $\mathrm{sign}(V) \times (eV/k_{\rm B}T_{\rm K})^2$ [Supplementary Figure 3(b)]. This may be attributed to the two-stage Kondo effect [6] and we do not use several points near zero bias to avoid this effect.

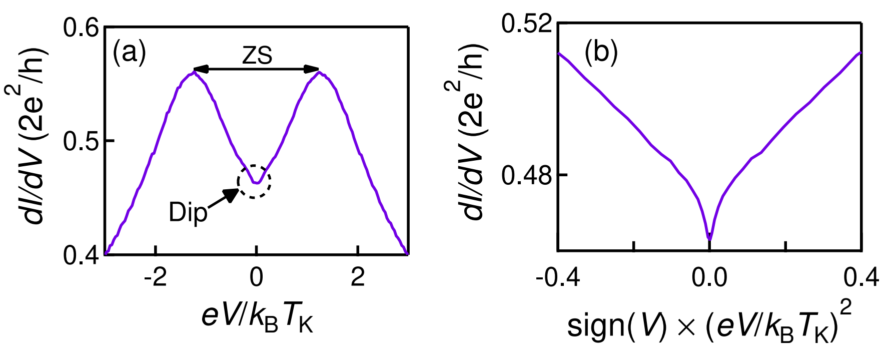

Supplementary Figure 3: (a) dI/dV as a function of $eV/k_{\rm B}T_{\rm K}$ at 2 T. The circle points out a very small dip or cusp-like structure just around the zero bias, which might be attributed to the two-stage Kondo effect. (b) dI/dV as a function of $\mathrm{sign}(V) \times (eV/k_{\rm B}T_{\rm K})^2$ at 2 T. The small dip in the left figure is attributed to the sharp slope around zero bias.

Supplementary Figures 4 (a) and (b) are $\mu_B B/k_B T_K$ and ε/U dependence of $R-1$, which are given by the NRG calculations with $U/\Gamma = 3.5$, respectively [5]. Here, R is the Wilson ratio. The Wilson Ratio decreases as the time-reversal and particle-hole symmetry are broken. The insets are the expanded views of $R-1$ whose horizontal scale corresponds to those shown in the main text, showing the value does not change in these regions. Thus, we use experimental value of $R = 1.95$ at zero field and at $\varepsilon = -U/2$ for our analysis.

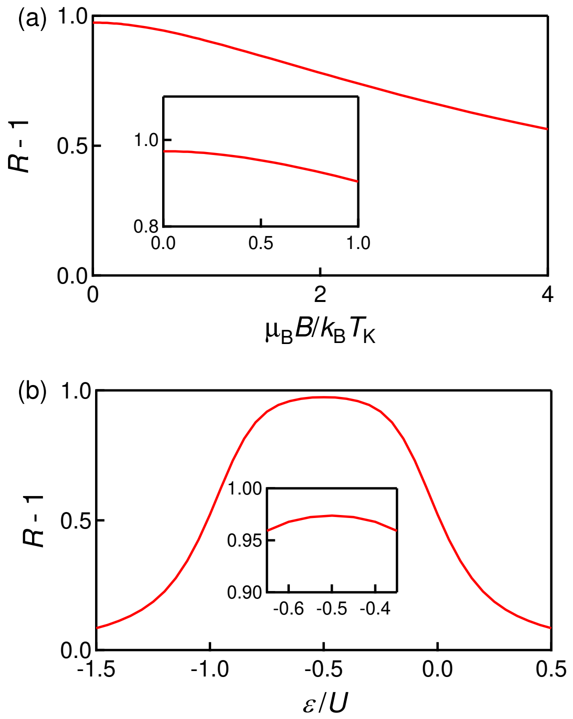

Supplementary Figure 4: (a) (b) $R-1$ as a function of $\mu_B B/k_B T_K$ and ε/U, respectively, which are given by the NRG calculations with $U/\Gamma = 3.5$ [5].

SUPPLEMENTARY NOTE 3

Analysis procedure

We show the analysis procedures to obtain W_2 and W_3 as a function of magnetic field and gate voltage as follows.

Magnetic field dependence of W_2

1. We obtain T_K by analyzing the temperature dependence of G_0 [7].
2. We obtain $T^*_K(\varepsilon = 0,\ B)$ by analyzing dI/dV at each field [Supplementary Note 2 and Supplementary Fig. 2(c)].
3. We derive $m_d(B)$ from $G_0(B)$ by using $G_0 = \frac{2e^2}{h}\cos^2(\pi m_d)$ (Fig. 3a in the main text).
4. We obtain $R = 1.95$ at $B = 0$ T by the shot noise measurement [7].
5. We assume that R is constant up to $\mu_B B/k_B T_K \sim 1$, which is justified by the NRG calculation [Supplementary Note 2 and Supplementary Fig. 4(a)].
6. We obtain W_2 as a function of $\mu_B B/k_B T_K$ by using $W_2 = -\left[1 + 5(R-1)^2\right]\cos(\pi m_d)$ (Fig. 3a in the main text).

Magnetic field dependence of W_3

1. We plot dI/dV as a function of $\mathrm{sign}(V) \times (eV/k_B T_K)^2$, and obtain α_V at each magnetic field by fitting the plotted points with $dI/dV = G_0 - \alpha_V(eV/k_B T_K)^2$ (Fig. 3b in the main text).
2. We calculate W_3 by using α_V, W_2, T_K, and T^*_K with Equation (6) in the main text (Fig. 3c in the main text).

Gate voltage dependence of W_2

1. We obtain $T^*_K(\varepsilon,\ B = 0)$ by analyzing the temperature dependence of G_0 at each gate voltage [Fig. 2a in the main text].
2. We derive $\delta_\sigma(\varepsilon)$ from $G_0(\varepsilon)$ by using $G_0 = \frac{2e^2}{h}\sin^2\delta_\sigma$. Here, we assume that $\delta_\sigma = \delta_\uparrow = \delta_\downarrow$ and that $\delta_\sigma > \pi/2$ and $\delta_\sigma < \pi/2$ for $\varepsilon/U < -0.5$ and $\varepsilon/U > -0.5$, respectively [Fig. 4a in the main text].
3. We assume that R is constant in a wide region $|\varepsilon/U + 0.5| < 0.15$, which is justified by the NRG calculation [Section C3 and Supplementary Figure 4(b)].
4. We obtain W_2 as a function of ε/U by using $W_2 = \left[1 + 5(R-1)^2\right]\cos 2\delta_\sigma$ [Supplementary Figure 5].

Gate voltage dependence of W_3

1. We plot dI/dV as a function of $\mathrm{sign}(V) \times (eV/k_B T_K)^2$, and obtain α_V at each gate voltage by fitting the plotted points with $dI/dV = G_0 - \alpha_V(eV/k_B T_K)^2$ [Fig. 4b in the main text].
2. We obtain W_3 by using W_2, T_K, T^*_K, and Eq. (3) shown in the main text [Fig. 4c in the main text].

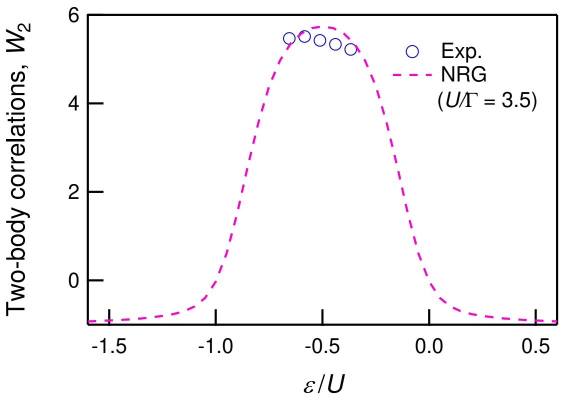

Supplementary Figure 5: W_2 as a function of ε/U. The points are experimental results, while the lines are theoretical ones.

SUPPLEMENTARY NOTE 4

Supplemental data

In this study, we measure the Kondo ridge at $N = 3$ in the sample that has been used in Refs. [7–9], where N is the number of electrons in the last shell. Supplementary Figure 6(b) is the color plot of dI/dV as a function of V and $V_{\rm g}$, where V and $V_{\rm g}$ are source-drain voltage and gate voltage, respectively. Here, the magnetic field, $B = 0.08$ T, is applied to suppress the superconductivity of the electrodes. The particle-hole symmetry point, $\varepsilon/U = -0.5$, is also indicated.

We also show W_2, m_d, $\alpha_{\rm V}$, and W_3 as a function of B in Supplementary Figures 7(a)–(c). While the same data as a function of the normalized magnetic field $\mu_{\rm B}B/k_{\rm B}T_{\rm K}$ are shown in Figs. 3a, 3b, and 3c in the main text, these graphs shown as a function of B itself might be useful for future analysis.

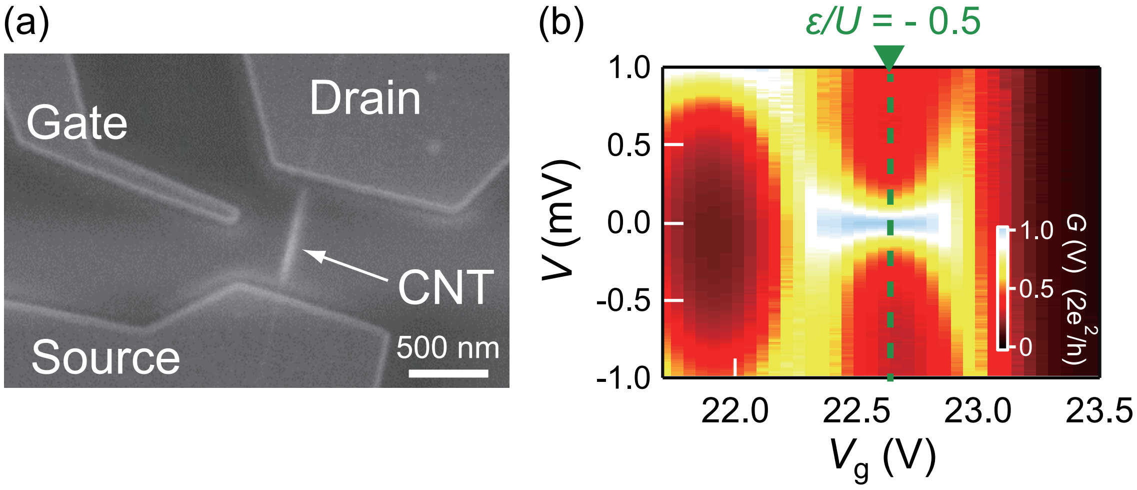

Supplementary Figure 6: (a) Scanning electron micrograph of a carbon nanotube quantum dot. (b) Color plot of dI/dV as a function of V and $V_{\rm g}$.

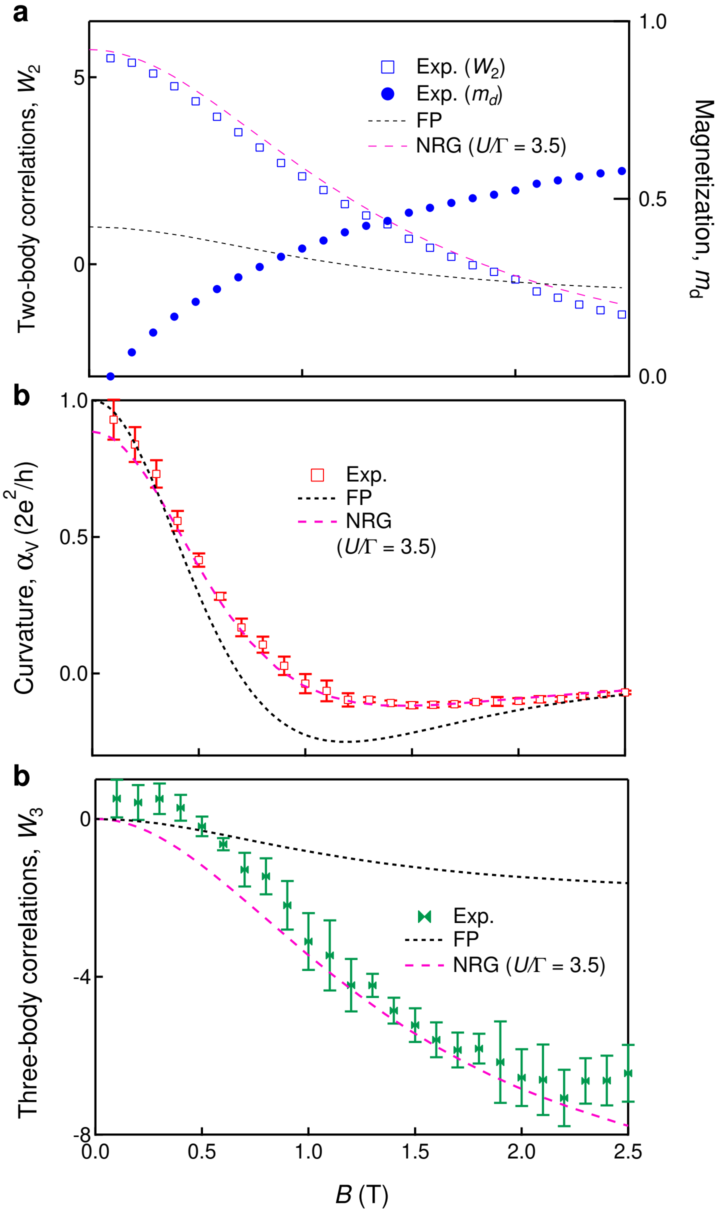

Supplementary Figure 7: (a) – (c) W_2, m_d, α_V, and W_3 as a function of magnetic field. The points are experimental results, while the lines are theoretical ones. The dotted and dashed lines for W_2 are given by the free particle (FP) model and the NRG calculations ($U/\Gamma = 3.5$), respectively. Error bars in Supplementary Figure 7(b) correspond to the uncertainty of the linear fit performed on slightly different ranges. The error bars in Supplementary Figure 7(c) are determined based on those of α_V shown in Supplementary Figure 7(b).

[1] Oguri, A. & Hewson, A. C. Higher-order Fermi-liquid corrections for an Anderson impurity away from half filling. *Physical Review Letters* **120**, 126802 (2018).

[2] Nozieres, P. A "Fermi-liquid" description of the Kondo problem at low temperatures. *Journal of Low Temperature Physics* **17**, 31–42 (1974).

[3] Yosida, K. & Yamada, K. Perturbation expansion for the anderson hamiltonian. *Progress of Theoretical Physics Supplement* **46**, 244–255 (1970).

[4] Oguri, A. Fermi liquid theory for the nonequilibrium Kondo effect at low bias voltages. *Journal of the Physical Society of Japan* **74**, 110–117 (2005).

[5] Oguri, A. & Hewson, A. C. Higher-order Fermi-liquid corrections for an Anderson impurity away from half filling: Nonequilibrium transport. *Physical Review B* **97**, 035435 (2018).

[6] van der Wiel, W. G. *et al.* Two-stage Kondo effect in a quantum dot at a high magnetic field. *Physical Review Letters* **88**, 126803 (2002).

[7] Ferrier, M. *et al.* Universality of non-equilibrium fluctuations in strongly correlated quantum liquids. *Nature Physics* **12**, 230–235 (2016).

[8] Ferrier, M. *et al.* Quantum fluctuations along symmetry crossover in a Kondo-correlated quantum dot. *Physical Review Letters* **118**, 196803 (2017).

[9] Hata, T. *et al.* Enhanced shot noise of multiple andreev reflections in a carbon nanotube quantum dot in SU(2) and SU(4) kondo regimes. *Physical Review Letters* **121** (2018).
